# PFAS Alter Thyroid Histology and Cellular Signaling *In Vitro* and *In Vivo*

**DOI:** 10.1210/jendso/bvaf210

**Published:** 2025-12-18

**Authors:** Heather A Hartmann, Kailey P Caroland, Goran W Tumbic, Jessica Rampy, Hua-Chang Chen, Sheau-Chiann Chen, Clara Mannes, Claudia C Wahoski, Matthew A Loberg, Cynthia Liang, Diana Diaz, Megan L Tigue, Quanhu Sheng, Fei Ye, Ethan Lee, Vivian L Weiss

**Affiliations:** Department of Pathology, Microbiology, and Immunology, Vanderbilt University Medical Center, Nashville, TN 37232, USA; Department of Pathology, Microbiology, and Immunology, Vanderbilt University Medical Center, Nashville, TN 37232, USA; Department of Cell and Developmental Biology, Vanderbilt University, Nashville, TN 37232, USA; Department of Pathology, Microbiology, and Immunology, Vanderbilt University Medical Center, Nashville, TN 37232, USA; Department of Biostatistics, Vanderbilt University Medical Center, Nashville, TN 37232, USA; Department of Biostatistics, Vanderbilt University Medical Center, Nashville, TN 37232, USA; Department of Pathology, Microbiology, and Immunology, Vanderbilt University Medical Center, Nashville, TN 37232, USA; Department of Pathology, Microbiology, and Immunology, Vanderbilt University Medical Center, Nashville, TN 37232, USA; Department of Pathology, Microbiology, and Immunology, Vanderbilt University Medical Center, Nashville, TN 37232, USA; Department of Pathology, Microbiology, and Immunology, Vanderbilt University Medical Center, Nashville, TN 37232, USA; Department of Pathology, Microbiology, and Immunology, Vanderbilt University Medical Center, Nashville, TN 37232, USA; Department of Pathology, Microbiology, and Immunology, Vanderbilt University Medical Center, Nashville, TN 37232, USA; Department of Biostatistics, Vanderbilt University Medical Center, Nashville, TN 37232, USA; Department of Biostatistics, Vanderbilt University Medical Center, Nashville, TN 37232, USA; Department of Cell and Developmental Biology, Vanderbilt University, Nashville, TN 37232, USA; Department of Pharmacology, Vanderbilt University, Nashville, TN 37232, USA; Department of Pathology, Microbiology, and Immunology, Vanderbilt University Medical Center, Nashville, TN 37232, USA; Department of Cell and Developmental Biology, Vanderbilt University, Nashville, TN 37232, USA; Department of Otolaryngology–Head & Neck Surgery, Vanderbilt University Medical Center, Nashville, TN 37232, USA

**Keywords:** PFAS, thyroid, toxicology, thyroid hormone, KLHL23, actin cytoskeleton

## Abstract

Perfluoroalkyl and polyfluoroalkyl substances (PFAS) are toxicants of emerging concern due to their abundance in the environment and potential adverse health effects. PFAS are used in waterproof clothing, makeup, carpets, upholstery, cookware, and fast-food containers. Due to their universal use, they are found globally in the water supply. In fact, these organic pollutants have been found in the blood of 98% of Americans and have been linked to disruption in thyroid hormone biosynthesis and availability. Moreover, several studies have shown that cancer patients may have an increase in PFAS levels and that PFAS exposure increases thyroid cancer risk. Demonstration of concrete PFAS-mediated alterations in thyroid histology and function could have far-reaching implications. To understand the effects of PFAS on thyroid histology, we used a PFAS feeding model with a combination of 3 PFAS compounds (PFOS, PFOA, and GenX) vs control. Mice receiving PFAS treatment showed altered thyroid architecture and cell structure following PFAS exposure. RNA-sequencing data revealed several alterations in gene expression and multiple signaling pathways were dysregulated. Understanding the role of PFAS-mediated toxicity in the thyroid is critically important for the prevention of thyroid disease in the general population.

Thyroid cancer incidence is on the rise, as it is predicted to be the fourth leading cause of cancer by 2030 (1-4). One of the reasons for this increase could be the increase in environmental pollutants, including endocrine-disrupting chemicals (EDCs). Perfluoroalkyl and polyfluoroalkyl substances (PFAS) are one type of EDC with a broad range of health effects, including thyroid hormone disruption and increased cancer risk (5, 6). After the discovery of Teflon, manufacturing of these chemicals began to take off, as PFAS started being used in many different products (7). Unsurprisingly, the highest contamination and exposures are found around their production and commercial manufacturers’ utilization sites (8-11). In addition to industrial sites, PFAS are also widely used in household products including makeup, carpets, and fast-food containers (10). Due to their high chemical and thermal stability, PFAS accumulate in the environment at high levels (10). While PFAS are not intended for consumption, these compounds are widely ingested through contaminated food and drinking water and can bioaccumulate in humans (12, 13). Altogether, the broad use of PFAS and resulting contamination has led to detectable levels of PFAS in the blood of 98% of Americans, despite efforts to monitor and reduce environmental PFAS levels (14-17).

Government regulation of “legacy” PFAS compounds, perfluorooctanoic acid (PFOA) and perfluorooctane sulfonic acid (PFOS) (18), has led to a rise in newer compounds, such as ammonium perfluoro(2-methyl-3-oxahexanoate) (GenX). Studies suggest that these newer compounds have increased cytotoxicity and genotoxicity, including in thyroid cells (19, 20). PFAS have also been shown to alter the biosynthesis and regulation of thyroid hormones in vitro and in silico (20-24), potentially through inhibition of thyrotropin (TSH) signaling and deiodinase activity, among other mechanisms. Other studies have shown that PFAS can alter thyroid hormone (both 3,5,3′-triiodothyronine [T_3_] and thyroxine [T_4_]) levels, leading to alterations in TSH levels in animal models and humans (18, 25). In addition, some epidemiological studies have linked thyroid cancer and PFAS exposure (26-28). To further investigate the link between PFAS and thyroid cancer, several *in vivo* studies showed that at high levels of PFAS exposure, animals develop thyroid follicular cell adenomas and nonneoplastic lesions (29, 30). Additionally, in other cancers, including kidney and testicular cancer, PFAS has also been shown to be correlated with higher levels of metastasis, especially in highly exposed populations (27, 31-33). A recent paper by Mei et al (31) showed that PFOA levels were increased in patients with lung adenocarcinoma and that PFOA increased metastasis in patients with lung adenocarcinoma through the reorganization of the cytoskeleton. However, we have limited knowledge of the mechanisms of PFAS-mediated thyroid disease.

In this study, we investigated whether exposure to 3 major PFAS compounds, PFOA, PFOS, and GenX, altered the histology and molecular profile of mouse and human thyroid. We used a PFAS feeding model to determine changes in thyroid histology in mice. We then assessed the molecular changes within these thyroids to investigate the possible mechanisms behind PFAS-mediated morphological changes. Of particular note, we found one gene that was significantly downregulated, *Klhl23*, which may explain structural alterations in thyrocytes after PFAS exposure, as the inhibition of KLHL23, a member of the Kelch-like family of proteins, has shown to lead to cytoskeleton alterations in urothelial and hepatocellular carcinoma (34, 35). This study presents a link between PFAS exposure and thyroid histology, providing mechanistic clues as to the role of PFAS in thyroid disease.

## Materials and Methods

### Perfluoroalkyl and Polyfluoroalkyl Substance Chemicals

PFOA was purchased from Sigma Aldrich (catalog No. 17168-5G). PFOS was purchased from Agilent Technologies (part No. N-0998). Ammonium perfluoro(2-methyl-3-oxahexanoate) (GenX) was purchased from Sigma Aldrich (MAN326369394).

### Cell Culture

Nthy-ori 3-1 cell line was obtained from Sigma Aldrich. Cells were authenticated using STR (short tandem repeat) analysis and maintained. Cells were grown in RPMI (VWR) containing 10% fetal bovine serum (FBS) (ThermoFisher Scientific), 1% penicillin-streptomycin (Sigma), 1× MEM nonessential amino acids (VWR), and 1-mM sodium pyruvate (Vanderbilt Molecular Biology Resource) (referred to as complete RPMI later).

### Murine Studies and Analysis

All procedures were approved by the Institutional Animal Care and Use Committee prior to completion (protocol M200095-01). 129S6/SvEv mice from Taconic Biosciences were used for these experiments. Mice were split into 2 groups (phosphate-buffered saline [PBS] n = 27 and PFAS n = 28) for each study. The PFAS-treated group was oral gavaged weekly with 7.5 mg/kg of each of 3 PFAS chemicals, PFOS, PFOA, and GenX, resuspended in PBS. The control group was oral gavaged with PBS weekly. Mice were assessed daily for appearance and behavioral changes and weighed weekly to ensure that weight loss did not exceed 20% of body weight. Mice were dosed weekly (8 doses total) and were euthanized after 8 weeks. Thyroids were resected from the mice after 8 weeks and placed in 10% neutral buffered formalin. Embedding, processing, and hematoxylin and eosin (H&E) staining of thyroids were performed by the Shared Pathology Resource at Vanderbilt University Medical Center. H&E images were analyzed using QuPath v0.4.4. Male (27 animals, 13 PBS and 14 PFAS) and female (28 animals, 14 PBS and 14 PFAS) mice were used in all experiments, and this was not considered a factor in the statistical analysis.

### RNA Sequencing

Five (5-µM width) sections of formalin-fixed, paraffin-embedded (FFPE) thyroid sample tissue were sectioned onto glass slides and sent to Vanderbilt Technologies for Advanced Genomics (VANTAGE) for processing and sequencing. All samples were sequenced (multiplex Paired-End 150 bp) on the Illumina NovaSeq 6000. Reads were trimmed to remove adapter sequences using Cutadapt (v4.8) (36). Quality control on both raw reads and adaptor-trimmed reads was performed using FastQC (v0.12.1) (37). Reads were aligned to the Gencode GRCm38.p6 genome using STAR (v2.7.11a) (38). Gencode vM25 gene annotations were provided to STAR to improve the accuracy of mapping. featureCounts (v2.0.6) (39) was used to count the number of reads mapped to each gene. ComplexHeatmap (40) was used for cluster analysis and visualization. Significantly differentially expressed genes (DEGs) with absolute fold change (FC) of 1.5 or greater and false discovery rate–adjusted *P* value of .1 or less were detected by DESeq2 (v1.42.1) (41) and visualized with R package EnhancedVolcano (v1.18) (42).

### Gene Set Enrichment Analysis and Overrepresentation Analysis

To investigate the functional enrichment of DEGs, we performed overrepresentation analysis (ORA) using WebGestaltR (v1.0.0) (43), and gene set enrichment analysis (GSEA) using the GSEAPreranked function in GSEA (v4.3.2) (44). For ORA, the DEGs were tested for significantly overrepresented pathways and Gene Ontology (GO) categories. GSEA was performed to identify gene sets that were significantly enriched in the ranked gene list, allowing the detection of subtle but coordinated changes across a gene set. The gene sets tested were collected from the KEGG database (45), GO database (46), and the Molecular Signatures Database (MSigDB, v2022.1.Hs) (47), specifically the Hallmark, Curated, and Ontology gene set collections.

### Gene Set Enrichment Analysis and Overrepresentation Analysis for Thyroid Hormone Signaling

Differential gene expression analysis was performed using DESeq2 to compare PFAS-treated and PBS control samples. Genes with an adjusted *P* value (*P*adj) less than .1 and absolute log2 FC (log2FC) greater than 1 were considered DEGs. We used this cutoff as there were not many differentially expressed thyroid hormone signaling–related genes. To investigate the potential effect of PFAS exposure on thyroid hormone signaling, we retrieved the gene set for the mouse thyroid hormone signaling pathway (KEGG pathway mmu04919). Two DEGs, *Atp1b2* and *Pfkm*, overlapped with this pathway. The KEGG pathway diagram was visualized using the pathview R package, with DEGs color-coded to reflect the direction and magnitude of expression changes (48). Additionally, we examined other thyroid hormone signaling–related genes, and these genes were highlighted in a volcano plot generated using the EnhancedVolcano package (42).

### Immunohistochemistry

Tissue sections (5-µm) were cut from FFPE blocks of PFAS and control mouse thyroids. Deparaffinization and antigen retrieval were performed as previously described for multiplex immunofluorescent staining of FFPE (49). After antigen retrieval, tissues were treated with BLOXALL endogenous peroxidase and alkaline phosphatase blocking solution (SP-6000-100, Vector Laboratories) for 10 minutes, washed with 0.05% Tween 20 in PBS, and blocked for 2 hours with 10% goat serum in PBS (blocking buffer). Primary antibody (rabbit anti-KLHL23 (SigmaAldrich, SAB1303309, RRID: AB_3675420) was diluted in blocking buffer 1:50 and incubated on tissue sections at 4 °C for 16 hours. Tissue sections were washed with 0.05% Tween 20 in PBS and incubated for 30 minutes with ImmPRESS HRP horse antirabbit immunoglobulin G polymer (MP-7801-15, Vector Laboratories). Tissue sections were washed with 0.05% Tween 20 in PBS and developed via incubation for 2 minutes with an equal mix of reagent 1 and reagent 2 of ImmPACT DAB EqV Substrate (MP-7801-15, Vector Laboratories). Following development, tissue sections were rinsed in distilled water, counterstained for 3 minutes with Mayer's Hematoxylin (MHS32-1L, MilliporeSigma), rinsed in water, washed for 1 minute in Scott's water (10 g magnesium sulfate, 2 g sodium bicarbonate, 1 L water), rinsed in water, dehydrated (1 minute each of 25% ethanol, 50% ethanol, 70% ethanol, and 100% ethanol, and 3 minutes xylene), and cover-slipped. Immunohistochemistry (IHC) staining was analyzed by a pathologist (VLW) for intensity and extent of staining. IHC strength and extent were multiplied, plotted in Prism (v10.5.0), and analyzed with a Mann-Whitney statistical test.

### Thyrotropin Multiplex Immunoassay Procedure

Blood was collected from 10 PBS- and 10 PFAS-treated mice via a cardiac stick (1-mL syringe with detachable 25-gauge needle, Becton Dickerson, SKU No. 309262) post mortem. Blood was transferred to plastic microcentrifuge tubes and sat at room temperature for 60 minutes to coagulate. Samples were spun down at 5000 rpm for 10 minutes at 4° C. Serum was transferred to a fresh microcentrifuge tube and stored at −80 °C. Milliplex kit number MPTMAG-49K was purchased from Millipore, and immunoassay was performed according to manufacturer's guidelines. Samples were not diluted, and fluorescence was detected with a Luminex MAGPIX in the Vanderbilt University Medical Center Analytical Services Core.

### Perfluoroalkyl and Polyfluoroalkyl Substance Cell Culture

Nthy-ori 3-1 cells were maintained in either control complete RPMI media or complete RPMI media with 100-µM PFOA, 100-µM PFOS, and 100-µM GenX for 8 weeks. Cells were passaged once a week in either control complete RPMI media or complete RPMI media with 100-µM PFOA, 100-µM PFOS, and 100-µM GenX during the 8 weeks.

### Small Interfering RNA–Mediated Knockdown

Nthy-ori 3-1 cells were transfected with KLHL23 siRNA (UGGAAGGCCUUGUAAAUUA, Dharmacon) using Lipofectamine 3000 reagent (Thermo Fisher Scientific) and following the manufacturer's protocols. After 72 hours of incubation, KLHL23 knockdown efficiency at 25, 50, and 100 nM small interfering RNA (siRNA) was monitored by Western blot analysis.

### Western Blot Analysis

Cells were lysed using nondenaturing lysis buffer (50-mM Tris-HCl pH 7.4, 300-mM NaCl, 5-mM EDTA, and 1% Triton X-100 (w/v)) supplemented with 1 mM PMSF and PhosSTOP phosphatase inhibitor cocktail tablets (Roche). Samples were gently agitated at 4 °C for 15 minutes, followed by clarification by spinning in a microfuge at 13 000 RPM for 10 minutes at 4 °C. Proteins were analyzed by sodium dodecyl sulfate–polyacrylamide gel electrophoresis. Fluorescence signal was detected using an Odyssey (LI-COR). Obtained images and band intensity were analyzed using Image Studio (LI-COR). The following antibodies were used: rabbit anti-KLHL23 antibody (1:500, Sigma-Aldrich, SAB1303309, RRID: AB_3675420), mouse anti-GAPDH (1:1000, Cell Signaling Technology catalog No. 97166, RRID: AB_2756824), goat antirabbit 800 (LI-COR, RRID: AB_2651127), and goat antimouse 680 (LI-COR, RRID: AB_2651128). All secondary antibodies were used at 1:20 000 dilution.

### Two-Dimensional Immunofluorescence

Immunofluorescence was performed as previously described in Lee et al (50). After approximately 8 weeks, 15 000 cells were plated into a 12-well plate onto glass coverslips in either fresh control complete RPMI media or fresh complete RPMI media with 100 µM of PFOA, 100-µM PFOS, and 100-µM GenX and allowed to attach for 48 hours. Cells were then fixed using 4% formaldehyde in a cytoskeleton buffer solution (10-mM MES pH 6.1, 138-mM KCl, and 2-mM EGTA plus sucrose (0.114 g/mL)). Cells were then washed with 0.1% TBST and permeabilized with 0.5% TBST for 10 minutes. Cells were then blocked in Abdil (0.1% TBST and 5% BSA) for 1 hour and washed with 0.1% TBST. Following blocking, cells were then stained with antiphalloidin 568 (1:1000, ThermoFisher No. A12380) and Hoescht (1:1000, ThermoFisher No. 66249) for 20 minutes. Cells were then washed for 10 minutes in 0.1% TBST and were mounted onto slides using ProLong Gold (Invitrogen). For siRNA-mediated knockdown, Nthy-ori 3-1 cells were seeded at a density of 8 × 10^4^ cells/mL onto coverslips coated with poly-D-lysine. Cells were transfected with 100 nM *Klhl23* siRNA for 48 hours, then fixed with 4% formaldehyde and permeabilized in 0.5% TBST. The samples were then blocked using Abdil and incubated with antiphalloidin 488 (1:1000, Cell Signaling Technology No. 8878). Samples were mounted in ProLong Gold with DAPI (4′,6-diamidino-2-phenylindole).

### Semi-Solid Spheroid Seeding and Maintenance

Nthy-ori 3-1 cells, K1 cells (purchased from Sigma-Aldrich), or MDA-T68 cells (purchased from ATCC) (not exposed to PFAS) were mixed with 5% Matrigel in complete RPMI media with 10% FBS. Cells were plated at 1000 cells/well in 550-μL complete RPMI media/Matrigel/FBS or complete RPMI media + 100-µM PFOA, 100-µM PFOS, and 100-µM GenX in each well of a 24-well ultra-low attachment plate (Corning 3473). Fresh complete RPMI media with either 10% FBS + 5% Matrigel or 10% FBS + 5% Matrigel + 100-µM PFOA, 100-µM PFOS, and 100-µM GenX was added dropwise to the culture once per week (3 times total). After 3 to 5 weeks of PFAS exposure, cells were imaged. K1 and MDA-T68 spheroid results were plotted in Prism 10.5.0 and analyzed with a Mann-Whitney statistical test. For the Nthy-ori 3-1 separate PFAS treatment conditions, a similar procedure was followed as described earlier, but instead of exposure to all 3 PFAS (PFOA, PFOS, and GenX), cells were exposed to 100 µM of GenX, 100 µM of PFOA, or 100-µM PFOS. Nthy-ori 3-1 separate PFAS treatment spheroid results were plotted in Prism 10.5.0 and analyzed with a Kruskal-Wallis statistical test.

### Brightfield Imaging and Immunofluorescence Imaging

Images of the Nthy-ori 3-1 spheroids and K1 spheroids were taken at 3 weeks following PFAS treatment. Images of the MDA-T68 spheroids were taken at 5 weeks following PFAS treatment. For each experimental group, 4 wells of control spheroids and 4 wells of PFAS-treated spheroids were imaged using brightfield microscopes (Leica DMi1 inverted microscope with 4× objective, EVOS FL inverted microscope with 10× objective, or EVOS FL inverted microscope with 20× objective). Images were taken at 4×, 10×, or 20× magnifications. Immunofluorescence imaging was performed using a CoolSNAP ES camera mounted on a Nikon Eclipse 80i fluorescence microscope with a 40× objective.

### Image Analysis

Images of the Nthy-ori 3-1 cells were analyzed using ImageJ to quantify spheroid size (area) and budding in semi-solid spheroids. Budding was measured as a binary variable (yes, indicating the presence of budding, or no, indicating its absence) for each spheroid. ImageJ was used to quantify the immunofluorescence, and relative filopodia and lamellipodia. For siRNA-treated cells, immunofluorescence quantification was performed across 9 different fields of view (each field of view had >10 cells). Perinuclear localization was similarly evaluated as a binary variable (yes, indicating perinuclear staining, or no, indicating its absence). Perinuclear localization results were plotted using Prism (v10.5.0) and analyzed with a Mann-Whitney statistical test.

### Statistical Analysis

Linear regression was used to evaluate the association between treatment and continuous outcomes, adjusted for potential batch effect from 3 experiments with 10 biological replicates each. We used this test for follicle area, colloid area, and nuclei count analyses. A linear mixed-effects model was used to evaluate the association between treatment and continuous outcomes to account for correlation among the technical replicates within a sample. We used this test for fluorescence intensity, relative filopodia, relative lamellipodia, and semi-solid spheroid size analyses. A general linear mixed-effect model was used to evaluate the association between treatment and budding (binary: no vs yes), where budding was assumed to follow a binomial distribution. This model accounts for the correlated observations from the same sample identification since we had 3 biological samples with multiple technical replicates within a sample for each treatment. The outcome by group was estimated using the least-squares means (model-based means), and the difference among groups was compared by the Wald test. Effects estimated with a *P* value less than .05 were considered statistically significant. All statistical analyses were conducted in R (v4.4.2) unless stated otherwise.

## Results

### *In Vivo* Perfluoroalkyl and Polyfluoroalkyl Substance Treatment Leads to Alteration in Thyroid Histology

**Figure 1.**
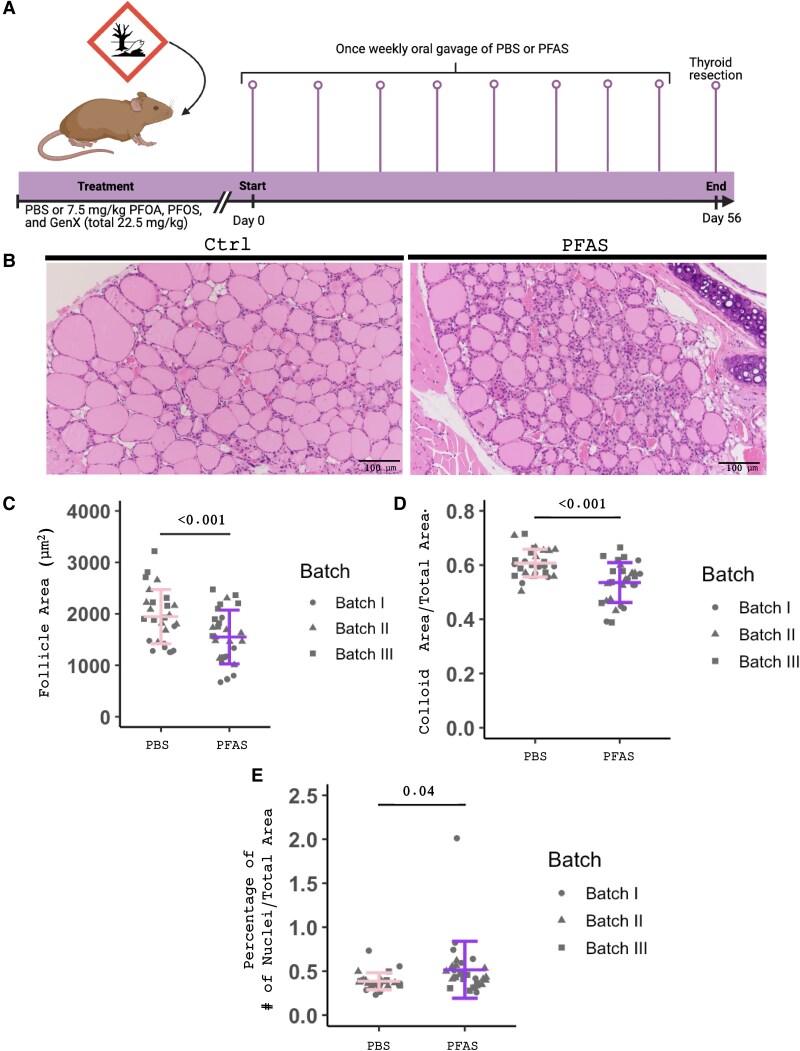
Perfluoroalkyl and polyfluoroalkyl substances (PFAS) treatment disrupts normal thyroid histology. A, Schematic of phosphate-buffered saline (PBS) or PFAS treatment. Created in BioRender. Hartmann, H. (2026) B, Hematoxylin-eosin stain (H&E) of PBS- and PFAS- treated mouse thyroid. Quantification of thyroids from H&E: C, Average follicle size in PBS vs PFAS treatment. D, Colloid area per thyroid area in PBS vs PFAS treatment. E, Cellularity area per thyroid in PBS vs PFAS treatment was calculated as a percentage of the number of nuclei to total thyroid area. Each symbol represents one thyroid, and batch represents 3 experiments. *P* values calculated using linear regression model.

Epidemiologic studies have suggested that high PFAS levels correlate with decreased thyroid hormone production; however, little is known about the direct effects of PFAS chemicals on thyroid histology and thyroid neoplasia. To answer this question, we treated mice with a mixture of 3 compounds, PFOA, PFOS, and GenX (7.5 mg/kg each, oral gavage), or PBS as a control every week for 8 weeks (Fig. 1A). The thyroids were resected at 8 weeks and H&E staining performed. Thyroid histology was evaluated by a trained pathologist with expertise in thyroid pathology. As seen in Fig. 1B, there is a striking difference in the size of the follicles between the PBS and PFAS groups (see Fig. 1B). Not only is there a change in follicle size, but there is prominent thyrocyte hypertrophy, with increased overall cell size, increased cytoplasm, and larger nuclei. Further quantification of the H&E staining of the thyroids of PFAS mice showed a decrease in follicle area (*P* < .001) and total colloid area to total area (*P* < .001) (Fig. 1C and 1D). We also counted the number of nuclei in each thyroid and compared that to the total area. We found an increase in cellularity (*P* = .04) compared to PBS control (Fig. 1E). In conclusion, we see that PFAS alters thyroid histology by decreasing follicle size, increasing cellularity, and promoting thyrocyte hypertrophy.

### *In Vivo* Perfluoroalkyl and Polyfluoroalkyl Substance Treatment Leads to Alteration in KLHL23

**Figure 2.**
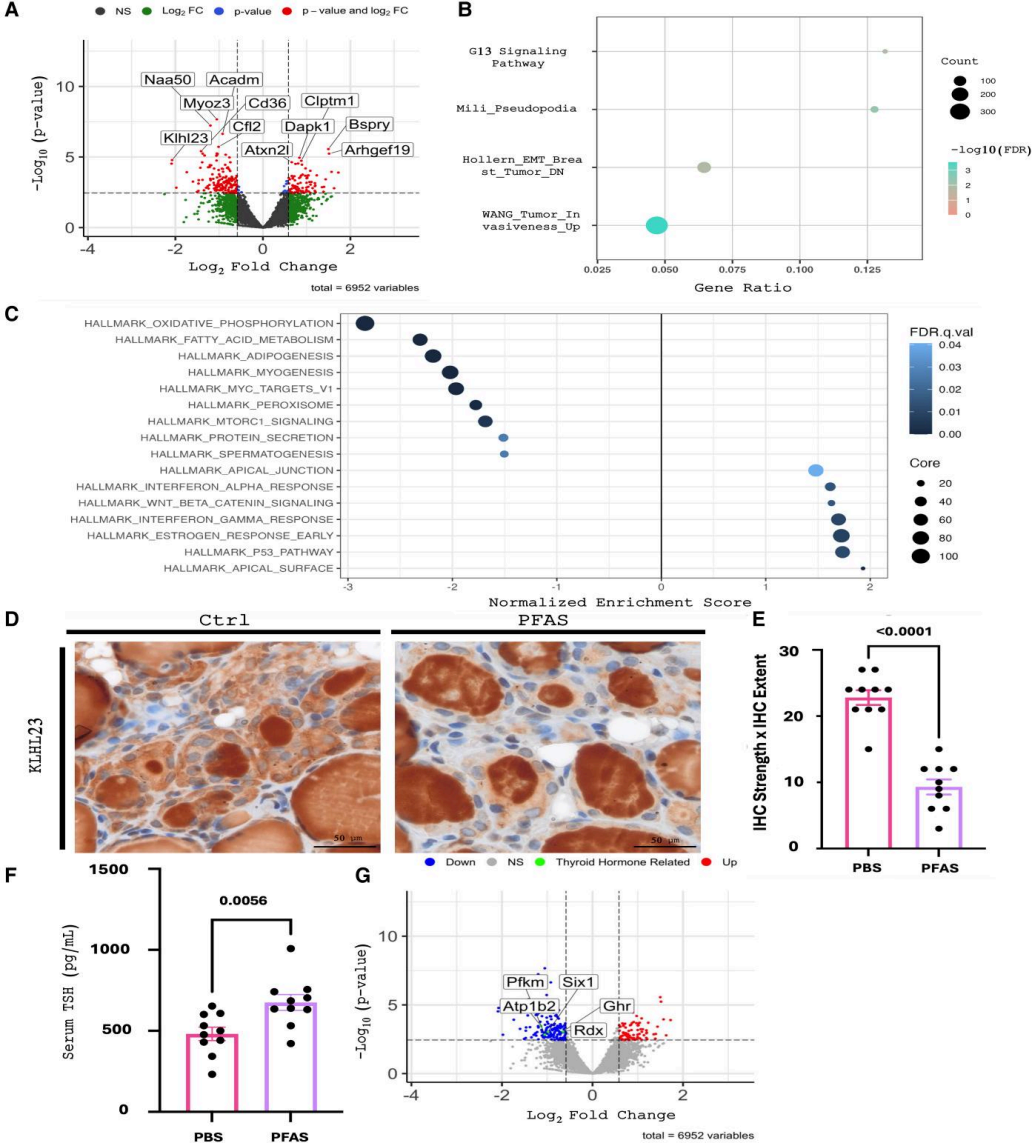
RNA sequencing shows altered gene expression in thyroids of perfluoroalkyl and polyfluoroalkyl substance (PFAS)-treated mice. A, Volcano plot of differentially expressed genes with an adjusted *P* value less than or equal to .1 and log_2_ fold change of 1.5 or greater. B, Overrepresentation analysis showing the most upregulated and downregulated pathways in phosphate-buffered saline (PBS)- vs PFAS-treated mice. C, Gene set enrichment analysis showing the most upregulated and downregulated pathways in PBS- vs PFAS-treated mice. D, Representative immunohistochemistry (IHC) of KLHL23 in a PBS (ctrl) and a PFAS mouse thyroid showing increased staining of follicular cells in control mice. E, Quantification of IHC staining strength and extent. *P* values calculated using Mann-Whitney. F, Serum thyrotropin levels (pg/mL) in PBS- and PFAS-treated mice. *P* values calculated using Mann-Whitney. 2G, Volcano plot of differentially expressed genes related to thyroid hormone signaling with an adjusted *P* value less than .1 and log_2_ fold change greater than 1.

To further understand the molecular mechanisms behind the PFAS-mediated changes in thyroid histology, we performed RNA sequencing on 8 mouse thyroids (4 PBS and 4 PFAS treated mice) to identify DEGs and differences in gene ontology (Supplementary Fig. S1) (51). We found the expression of several genes, including *Dapk1*, *Bspry*, and *Clptm1*, to be the most upregulated in our PFAS samples, as well as *Cd36*, *Naa50*, *Myoz3*, and *Klhl23*, to be the most downregulated (Fig. 2A). In addition, GSEA and ORAs identified several different cellular pathways that were enriched in the sequencing data. We found that G13 signaling, pseudopodia, epithelial-to-mesenchymal transition (EMT) breast cancer invasiveness, and tumor cell invasiveness were all significantly enriched in our PFAS mice (Fig. 2B and Supplementary Fig. S2) (51). Additionally, several hallmark pathways were enriched in our PFAS samples, including Wnt signaling (Fig. 2C). Overall, these RNA sequencing results support changes in the actin cytoskeleton as well as activation of several pro-tumorigenic signaling pathways.

Given the increased thyrocyte size following PFAS treatment and the changes in gene expression related to the actin cytoskeleton, we next wanted to further evaluate decreased *Klhl23* expression in the mouse thyroid. KLHL23 is known to bind to actin and suppress actin polymerization, thereby suppressing actin remodeling and promoting cell adhesion. In urothelial and hepatocellular carcinoma, reduced expression of KLHL23 was shown to influence actin stress fibers, increasing cell motility, and promoting EMT (34, 35). We confirmed protein expression of KLHL23 in our mouse thyroids, with and without PFAS treatment, using IHC. In agreement with the PFAS-mediated drop in *Klhl23* gene expression identified by RNA sequencing, we identified decreased cytoplasmic protein expression of KLHL23 in PFAS-treated mice (*P* < .0001; Fig. 2D and 2E). To summarize, we used RNA sequencing and immunohistochemistry to reveal that KLHL23 is significantly downregulated in PFAS-treated mouse thyroids.

### *In Vivo* Perfluoroalkyl and Polyfluoroalkyl Substance Treatment Leads to Alterations in Thyrotropin and Thyroid Hormone Signaling

Within our RNA sequencing data, we did not originally observe any statistically significant changes in gene expression related to thyroid hormone biosynthesis or signaling. This was unexpected because the hypertrophy and increased cellularity that we observed histologically are well-known effects of elevated TSH (52), which should promote thyroid hormone biosynthesis-related gene expression. Additionally, it has been well established that PFAS alter thyroid hormones both in humans and animals (53-55). To explore this further, we measured serum TSH levels both in our PBS control and PFAS-treated mice and found that the PFAS-treated mice displayed elevated TSH levels (*P* = .0056) (Fig. 2F). We also used a different threshold to analyze our RNA sequencing data ([log2FC] > 1), identifying several thyroid hormone–related genes (*Atp1b2*, *Ghr*, *Six1*, *Rdx*, and *Pfkm*) that were downregulated (Fig. 2G). Two of these genes, *Atp1b2* (ATPase Na^+^/K^+^ Transporting Subunit Beta 2) and *Pfkm* (phosphofructokinase), are annotated within the KEGG mouse thyroid hormone signaling pathway (Supplementary Fig. S3) (51). *Atp1b2* encodes a subunit of the Na^+^/K^+^-ATPase. This gene is a classic thyroid hormone–signaling target, and it is known to modulate the sodium gradient that is critical for the sodium/iodide symporter to concentrate iodide into the thyroid for thyroid hormone biosynthesis (56, 57 ). Its downregulation suggests both that thyroid hormone levels may be low and that thyroid hormone biosynthesis may be reduced. *Pfkm*, the muscle type isoform of phosphofructokinase, is likely derived from the surrounding muscle present in the bulk RNA sequencing sample. It is a known thyroid hormone–signaling target included in the KEGG pathway (see Supplementary Fig. S3) (51), and its downregulation is thus consistent with decreased thyroid hormone levels. Additionally, *Ghr* has been shown to be expressed by thyroid cells and is regulated by thyroid hormones but not TSH, so its decrease supports lower thyroid hormone levels specifically (58-60). Finally, the 2 other DEGs, *Six1* and *Rdx,* are included in GO pathways related to the cellular response to thyroid hormones, although more work needs to be conducted to characterize their regulation by thyroid hormones. Interestingly, thyroid hormone biosynthesis gene expression was not significantly upregulated, even in this second analysis, despite elevated TSH levels, which could suggest impairment of thyroid hormone biosynthesis downstream of TSH signaling. In conclusion, we demonstrate that PFAS treatment led to elevated TSH levels and moderate changes in thyroid hormone signaling, which suggests decreased thyroid hormone levels.

### *In Vitro* Long-Term Exposure to Perfluoroalkyl and Polyfluoroalkyl Substances Alters Actin Stress Fibers

**Figure 3.**
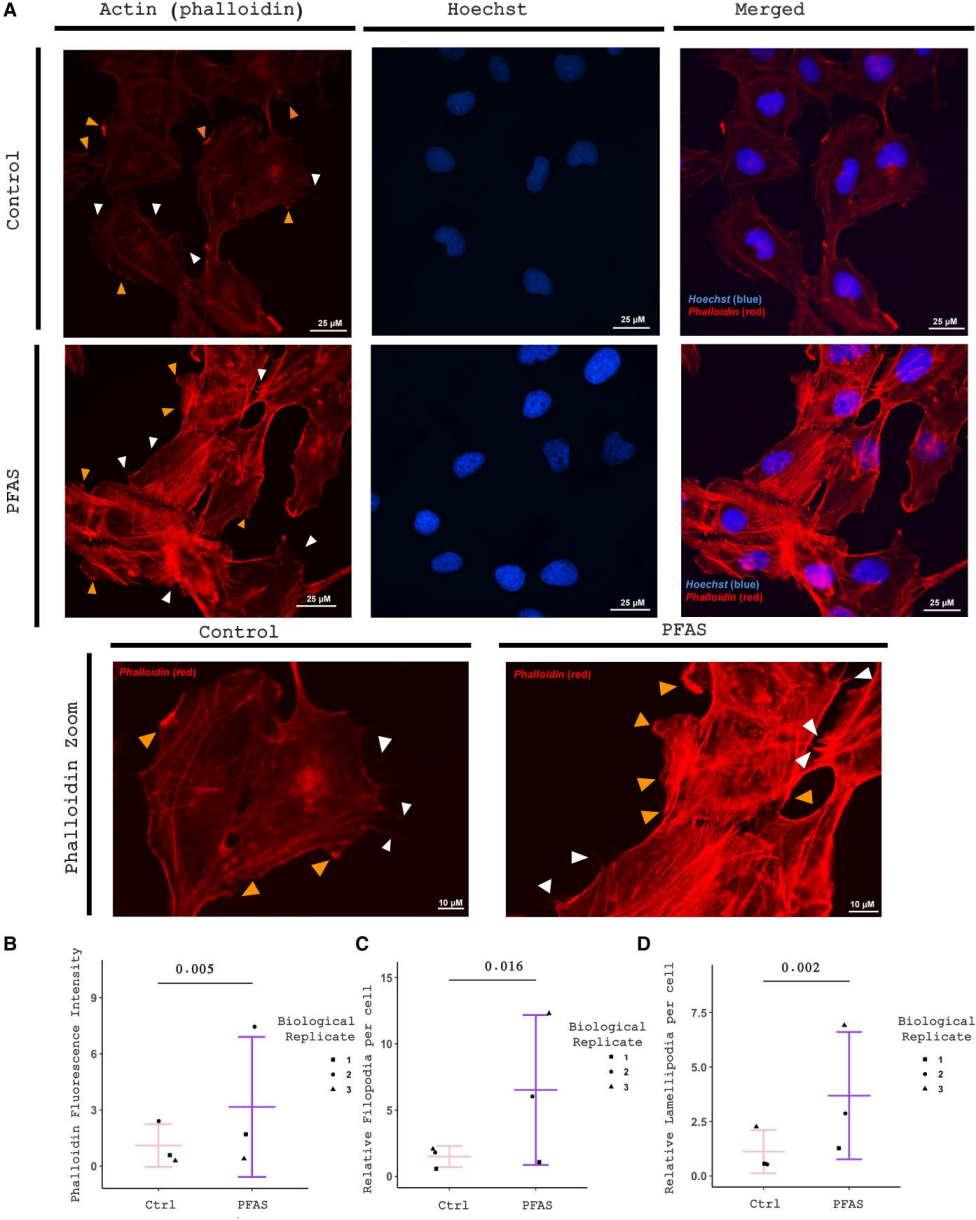
Actin stress fibers are altered in perfluoroalkyl and polyfluoroalkyl substance (PFAS)-treated Nthy-ori 3-1 cells. A, Actin immunofluorescence staining of control (media-treated) Nthy-ori 3-1 cells and PFAS-treated Nthy-ori 3-1 cells for approximately 8 weeks. Filopodia (white arrows) and lamellipodia (orange arrows) are also shown at higher magnification. B, Quantification of immunofluorescence staining intensity in control vs PFAS-treated Nthy-ori 3-1 cells. C, Quantification of relative filopodia in control vs PFAS-treated Nthy-ori 3-1 cells. D, Quantification of relative lamellipodia in control vs PFAS-treated Nthy-ori 3-1 cells. *P* values calculated using linear mixed-effects model.

To further confirm the role of PFAS treatment on cellular structure and actin polymerization, we performed immunofluorescence staining for phalloidin (F-actin) on Nthy-ori 3-1 cells treated for approximately 8 weeks with either media or 100-µM PFOA, PFOS, and GenX. Importantly, Nthy-ori 3-1 cells lack functional thyroid hormone synthesis and secretion, and this culture model did not include TSH addition to the media. Using this system allows for an evaluation of PFAS-mediated effects independent of thyroid hormone and TSH signaling. We found an increase in actin stress fiber formation as quantified using ImageJ (*P* = .005) (Fig. 3A and 3B), in line with the PFAS-mediated reduction in KLHL23 expression we found in our mouse model. To further understand the role that increased actin stress fibers may be playing in our cells, we also measured filopodia and lamellipodia, which are known markers of cell motility. We found that PFAS treatment increased both filopodia (*P* = .016) and lamellipodia (*P* = .002) (Fig. 3C and 3D).

To further explore the mechanism behind the actin cytoskeleton alterations, we performed an siRNA-mediated short-term knockdown of *Klhl23* in our Nthy-ori 3-1 cells and stained for phalloidin (F-actin) (Supplementary Fig. S4A and S4B) (51). We identified striking changes in the actin cytoskeleton, which included strong perinuclear actin localization in the knockdown cells (*P* = .0005) (Supplementary Fig. S4C) (51). Further investigation is needed to explore the relationship between KLHL23, PFAS, and actin fiber formation.

### In Vitro Long-Term Exposure to Perfluoroalkyl and Polyfluoroalkyl Substances Alters Spheroid Size

**Figure 4.**
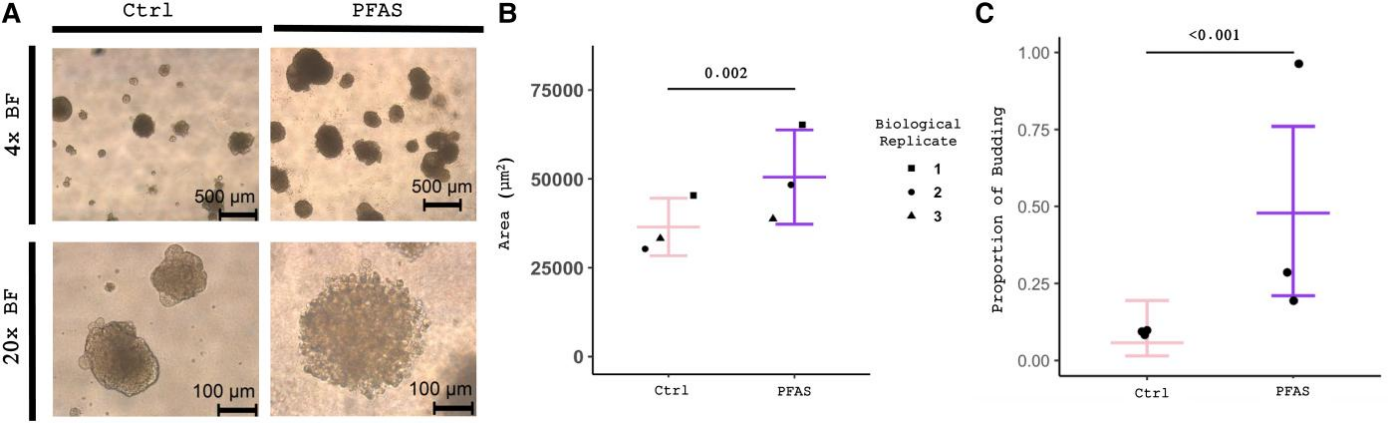
Altered size and morphology in perfluoroalkyl and polyfluoroalkyl substance (PFAS)-treated spheroids. A, Brightfield images of control (media-treated) Nthy-ori 3-1 cells and PFAS-treated Nthy-ori 3-1 cells for 3 weeks. B, Quantification of spheroid size in control vs PFAS-treated Nthy-ori 3-1 cells. C, Quantification of estimated proportion of budding spheroids in control vs PFAS-treated Nthy-ori 3-1 cells with 95% asymptotic confidence interval. *P* values calculated using model-based mean and CI.

In addition to analysis of 2-dimensional cell culture, we wanted to investigate how PFAS treatment altered 3-dimensional spheroid formation in a culture system that lacked significant thyroid hormone and TSH signaling. We plated Nthy-ori 3-1 cells using our previously published semi-solid spheroid methodology (50) with and without PFAS treatment (100-µM PFOA, 100-µM PFOS, and 100-µM GenX). We found that after 3 weeks, the PFAS spheroids were larger compared to controls (*P* = .002) (Fig. 4A and 4B). Additionally, we quantified the proportion of spheroids in each treatment group that were budding and found that our PFAS-treated spheroids showed an increase in buds (*P* < .001) (Fig. 4C). This budding morphology was similar to what we observed in our thyroid cancer spheroids previously (50).

To further explore these changes, we wanted to pinpoint which PFAS compound was responsible for the increase in size and budding. We plated Nthy-ori 3-1 cells with and without individual PFAS treatment (either 100-µM GenX, 100-µM PFOA, or 100-µM PFOS). We found that after 3 weeks, the GenX (*P* = .0006) and PFOS (*P* < .0001) spheroids were significantly larger compared to controls (Supplementary Fig. S5A and S4B) (51 ). We also observed a trend for PFOA to increase spheroid size compared to controls (*P* = .0539) (see Supplementary Fig. S5A and S4B) (51). Additionally, we quantified the proportion of spheroids in each treatment group that were budding and found that our PFOS-treated spheroids showed the highest increase in buds with approximately 89.2% of all spheroids showing budding. GenX and PFOA resulted in 71.5% and 42.5% of the spheroids budding, respectively, and control spheroids exhibited 59.8% budding. In conclusion, PFAS treatment of Nthy-ori 3-1 spheroids leads to an increase in spheroid size with PFOS showing the highest increase. The results suggest similar morphologic effects on thyrocytes with each compound, particularly PFOS and GenX. Importantly, given that TSH is absent in this culture model, the morphologic changes identified are direct effects of PFAS on thyrocytes, independent of alterations in TSH levels or signaling.

Finally, we wanted to explore the role of PFAS treatment on thyroid cancer spheroids. To study this, we used 2 thyroid cancer cells lines, MDA-T68 (a follicular variant of papillary thyroid cancer line with an activating *HRAS* Q61 K mutation) and K1s (a papillary thyroid cancer line with an activating *BRAF* V600E mutation). We plated MDA-T68 or K1 cells with and without PFAS treatment (100-µM PFOA, 100-µM PFOS, and 100-µM GenX). We found that after 5 weeks the MDA-T68 PFAS spheroids were larger compared to controls (*P* < .001) (Supplementary Fig. S5C and S5D) (51). In contrast, we found that after 3 weeks of treatment the K1 PFAS spheroids were smaller compared to controls (*P* < .001) (Supplementary Fig. S5E and S5F) (51). We did not measure budding because K1 spheroids, and to a lesser extent MDA-T68, normally exhibit budding morphology. The difference in cellular response and morphology likely reflects the differing biology and signaling processes occurring in these 2 different cancer types.

## Discussion

To date, there have been several studies that show PFAS alter thyroid physiology. However, little is known about the role of PFAS on thyroid histology and their possible connection to neoplasia. The goal of this study was to assess the effects of long-term PFAS exposure on thyroid histology and gene expression. Our *in vivo* findings show that PFAS chemicals decrease thyroid follicle size, increase thyroid cellularity, and lead to thyrocyte hypertrophy. We further discovered several differentially expressed genes and signaling pathways using RNA sequencing, including *Klhl23* and regulation of the actin cytoskeleton. We confirmed that PFAS treatment of mice decreased KLHL23 protein expression. We further confirmed that TSH levels were increased after PFAS treatment in mice, although interestingly, this did not lead to increased thyroid hormone biosynthesis gene expression or thyroid hormone signaling. Rather, our results suggest that thyroid hormone levels and signaling may be decreased. Our *in vitro* findings in a system without significant thyroid hormone or TSH signaling show that PFAS chemicals increase actin stress fibers, filipodia, and lamellipodia, potentially enhancing cell motility. We further used a 3-dimensional culture model to show an alteration in thyroid spheroid size and budding with PFAS treatment. Thus, PFAS may play an additional role altering thyrocyte size, motility, and gene expression in a TSH-independent manner.

This research enhances our understanding of PFAS-mediated thyroid disruption by providing a key finding that PFAS alter thyrocyte size, cellular organization, growth, and gene expression. The structure of thyroid follicles is key to the production of thyroid hormones, which are essential for the development and metabolism of many organisms. Without proper thyroid structure, the ability of this gland to efficiently produce and secrete thyroid hormones may be compromised, which could lead to reduced thyroid hormone levels or increased T_3_/T_4_ ratio (61). Our findings are in line with a struggling thyroid, which pairs nicely with the known changes in serum thyroid hormone levels following long-term PFAS exposure. For instance, several recent epidemiological studies have highlighted that PFAS exposure, including through drinking water, elevates the serum T_3_/T_4_ ratio. An elevated serum T_3_/T_4_ ratio could reflect changes in peripheral regulation of thyroid hormones, inadequate thyroid function, or both, and is seen in several forms of hypothyroidism, which could result in decreased thyroid hormone availability and action in target tissues (61-65). Finally, PFAS exposure has been shown to increase thyroid cancer risk (28, 53, 54, 66).

Although we did not observe many changes in thyroid hormone signaling–related gene expression after PFAS exposure, this was not entirely surprising. There are many variables to consider with gene expression analysis. For instance, our mice were treated for 8 weeks with PFAS, whereas other published *in vitro* and *in vivo* exposure models report gene expression changes starting at just 24 hours to 28 days (18, 19, 67). Time and dosing may indeed modulate the effects of PFAS on thyroidal gene expression. Another factor that could explain the moderate changes in thyroid hormone signaling is that our bulk RNA sequencing was performed in thyroid tissue, which is not itself a well-characterized target tissue of thyroid hormones. Moreover, there is considerable heterogeneity among tissues in terms of genes that respond to thyroid hormones (68 ). Thus, the available gene set annotations may not accurately and fully capture genes regulated by thyroid hormones in thyrocytes, and although we did identify several DEGs suggestive of reduced thyroid hormone availability and signaling, gene expression in thyroid tissue may not reflect systemic thyroid status. Further studies examining thyroid hormone signaling in the brain and metabolic tissues are needed to better understand the effects of PFAS on systemic thyroid hormone bioavailability and action, especially considering that thyroid hormone activation and turnover could also be altered in this model. Our findings in thyroid tissue do highlight a surprising lack of upregulation of the TSH-responsive genes involved in thyroid hormone biosynthesis. Further study is needed to identify the underlying mechanisms of PFAS-mediated thyrocyte functional impairment.

We also highlight a potential role of PFAS chemicals in altering signaling pathways central to tumorigenesis. While none of our mice developed tumors, we identified enhanced cell signaling consistent with EMT (69 , 70 ). In particular, our RNA sequencing and protein expression data show a decrease in KLHL23 expression, an actin regulator, after PFAS exposure. This finding is in line with several other studies that have shown alterations in KLHL23 in human cancer tissue compared to normal, including in thyroid cancer (71). Our *in vitro* studies also support a role for PFAS in altering actin cytoskeletal dynamics. KLHL23 is a promising mechanistic candidate for the effects of PFAS on thyroid structure, as its knockdown results in dramatic rearrangement of the actin cytoskeleton. The fact that knockdown of KLHL23 does not precisely phenocopy the changes in the actin cytoskeleton structures we observed in our PFAS-treated cells is not surprising. We have previously shown that alterations in growth pathways can result in dramatic changes in the actin cytoskeleton of thyroid cancer cells (50), and PFAS treatment is likely to affect several signaling pathways. Further studies are needed to determine the role that KLHL23 and other signaling pathways may play in the progression from normal thyroid tissue to thyroid cancer, as well as the regulation of actin stress fibers in the thyroid.

There are several key limitations of this study. First, our PFAS treatment is at high concentrations (a total of 22.5 ppm) and for only 8 weeks, which may not completely recapitulate the amount of PFAS in human blood and our constant prolonged exposure. The use of PFAS compounds in research studies varies dramatically, as it is challenging to fully model the varied exposure in humans. Some studies treated with 300 ppm for 2 years in rats (29), while others used 100 µM in vitro for approximately 3 weeks (31), a concentration similar to what we used in our study. Additional studies will be needed with lower concentrations and longer treatment durations to better represent the chronically accumulating levels of PFAS in humans. In addition, the use of cell lines in culture does not fully recapitulate endogenous thyrocytes and their physiology. However, despite the limitations of cell lines in culture, our *in vitro* findings closely mirror those identified in our mouse model.

### Conclusion

PFAS are known global contaminants with many roles in human health. In this study, we were able to confirm an alteration in mouse thyroid histology and an increase in TSH after PFAS treatment, which may signify impaired thyroid function. We also confirmed a decrease in KLHL23 using RNA sequencing and IHC after PFAS treatment. Finally, we observed alterations in actin stress fibers as well as alterations in spheroid formation. Taken together, we propose that decreased KLHL23 alters cell structure and behavior after PFAS treatment in ways that are predicted to be protumorigenic. More work needs to be conducted to ascertain the mechanism behind the decrease in KLHL23 and its possible role in thyroid cancer. More broadly, additional research is needed to further understand the role of PFAS and other EDCs on human health.

## Data Availability

Original data generated and analyzed during this study are included in this published article or in the data repositories listed in “References.” All data and materials will be shared by the lead contact (V.L.W.). If the lead contact should leave the institution, collaboration requests should be directed to the Pathology Department Chair (Dr Alice Coogan, alice.coogan@vumc.org). Code for all analyses is available at the following link on GitHub: https://github.com/chc-code/P11636_mouse_RNAseq.

## Abbreviations

DEGs: differentially expressed genes
EDCs: endocrine-disrupting chemicals
EMT: epithelial-to-mesenchymal transition
FBS: fetal bovine serum
FC: fold change
FFPE: formalin-fixed paraffin-embedded
GenX: ammonium perfluoro(2-methyl-3-oxahexanoate)
GO: Gene Ontology
GSEA: gene set enrichment analysis
H&E: hematoxylin and eosin
IHC: immunohistochemistry
ORA: overrepresentation analysis
PBS: phosphate-buffered saline
PFAS: perfluoroalkyl and polyfluoroalkyl substances
PFOA: perfluorooctanoic acid
PFOS: perfluorooctane sulfonic acid
siRNA: small interfering RNA
T_3_: 3,5,3′-triiodothyronine
T_4_: thyroxine
TSH: thyrotropin
